# A Penalized Linear and Nonlinear Combined Conjugate Gradient Method for the Reconstruction of Fluorescence Molecular Tomography

**DOI:** 10.1155/2007/84724

**Published:** 2007-08-27

**Authors:** Shang Shang, Jing Bai, Xiaolei Song, Hongkai Wang, Jaclyn Lau

**Affiliations:** Medical Engineering and Health Technology Research Group, Department of Biomedical Engineering, Tsinghua University, Beijing 100084, China

## Abstract

Conjugate gradient method is verified to be efficient for nonlinear optimization problems of large-dimension data. In this paper, a penalized linear and nonlinear combined conjugate gradient method for the reconstruction of fluorescence molecular tomography (FMT) is presented. The algorithm combines the linear conjugate gradient method and the nonlinear conjugate gradient method together based on a restart strategy, in order to take advantage of the two kinds of conjugate gradient methods and compensate for the disadvantages. A quadratic penalty method is adopted to gain a nonnegative constraint and reduce the illposedness of the problem. Simulation studies show that the presented algorithm is accurate, stable, and fast. It has a better performance than the conventional conjugate gradient-based reconstruction algorithms. It offers an effective approach to reconstruct fluorochrome information for FMT.

## 1. INTRODUCTION

Light with wavelength in the near-infrared range can propagate a few centimeters through the tissue because of low tissue absorption in the spectral of “near-infrared window.” This finding has encouraged the development of fluorescence techniques to visualize specific biochemical events inside living subjects [1, 2]. In recent years, a great development has happened to the fluorescence molecular tomography (FMT), a technique that resolves molecular signatures in deep tissue using fluorescent probes or markers [1, 3–6]. Tissue is illuminated by a series of excitation light in FMT; multiple measurements for the fluorescent emission light are collected from the tissue surface to resolve and quantify fluorochromes deep inside the tissue. With great potential, FMT has become a promising imaging modality for in vivo small animal imaging [1, 2].

Several reconstruction approaches for FMT have been proposed. Most of them are based on the diffusion model [6–10]. The model can be solved by methods such as finite difference method [8], finite element method [6], adaptive finite element method [11], and statistical method [12]. A weighting matrix can be obtained from the forward model, which describes the influence of each volume element on the detector readings. Generally, the inverse reconstruction problem of FMT is to find the fluorescent source distribution in the target tissue based on the precalculated weighting matrix and the measured data. Since the data measured from the tissue surface is far less than the number of unknown points inside the tissue, the reconstruction problem is illposed, and the solution is sensitive to noise as well as measurement error. Several algorithms have been reported, such as the modified Newton method-based optimization scheme [13] and the Born-type approximation techniques [14]. The conjugate gradient (CG) methods, which need less storage and computation, are favorable for the problems with large-dimension data. They have been reported to be adopted successfully in the reconstruction algorithms for imaging modalities such as the positron emission tomography (PET) [15–17] and diffusion optical tomography (DOT) [18]. Normally, two different kinds of CG with different properties are being used under different conditions. They are the linear CG method (L-CG) and the nonlinear CG method (N-CG) [19]. There is a remarkable point that L-CG and N-CG have reciprocal properties. Combining them together may generate an improved algorithm, which has the advantages of both of them.In this paper, a penalized linear and nonlinear combined conjugate gradient method (PLN-CG) for the reconstruction of FMT is presented. The L-CG method and the N-CG method are employed separately at different period based on a restart strategy, in order to exert their advantages while compensating for their disadvantages. Besides, a quadratic penalty method is adopted to give the result a nonnegative constraint, as well as reduce the uncertainty and illposedness of the problem. Simulation studies show that the PLN-CG algorithm can give a more accurate and more stable result for the reconstruction in FMT with less computation. Detailed description of the PLN-CG algorithm can be found in Section 3. Section 2 gives a general review of the forward and inverse problems in FMT, including the conventional CG-based reconstruction method. Simulation experiments are presented in Section 4 to demonstrate the validity and efficiency of the proposed algorithm. Section 5 summarizes the main results and gives a general discussion.

## 2. THEORY AND BACKGROUND

### 2.1 Forward model in FMT

When an external excitation light source works at continuous wave mode (CW mode), the following diffusion equation can be employed to model the propagation of the excitation light and the fluorescent emission light [6–10]:

(1) ∇ ⋅ [D_*x*_(**r**)∇Φ_*x*_(**r**)] − [*μ*_*ax*_(**r**) + *μ*_*af*_(**r**)]Φ_*x*_
(**r**) = −Θ_*s*_*δ* (**r**−
**r**_*sk*_), 
∇ ⋅ [D_*m*_(**r**)∇Φ_*m*_(**r**)] − *μ*_*am*_(**r**) Φ_*m*_(**r**) = −Φ_*x*_(**r**)
*ημ*_*af*_(**r**),

where **r** is the position vector belonging to the image region Ω. Φ*_x,m_*(**r**) represents the photon density at **r** for the excitation light (subscript *x*) or the fluorescent emission light (subscript *m*). *D*_*x,m*_(**r**) is defined as the diffusion coefficient

(2) *D*_*x,m*_(**r**) = (3 (*μ*_*ax,m*_(**r**) + (1 − *g*)*μ*_*sx,m*_(**r**)))^−1^ ,

where *μ*_*a*__*x*_,_*m*_(**r**) and *μ*_*sx*_,*_m_*(**r**) are the absorption and scattering coefficients, respectively. *g* is the anisotropy parameter. The absorption of the excitation light due to fluorophores is described as *μ* *_a_*_*f*_(**r**) and the fluorescent yield *η* *μ*_*a*__*f*_(**r**) is required for fluorescence parameter.

### 2.2. The inverse reconstruction problem in FMT

In this work, the finite element method is used to solve the forward model. Detailed description of the finite element method for the FMT forward problem can be found in [6, 11]. Based on the finite element solution of the forward problem, (1) is transformed into a linear matrix equation as follows:

(3) **Wx** = **I**,

where **x**, an *N* × 1 vector, denotes the real fluorescent source distribution to be reconstructed. **I**, a *M* × 1 vector, is the emission data computed from the measurement at the surface of the tissue. And **W**, a *M* × *N* matrix, is the weighting matrix generated from the forward model. Generally, the inverse problem for FMT is to find the fluorescent source distribution **x** in the target tissue from the measured data **I** and the precalculated matrix **W**. As mentioned before, the problem in (3) is quite illposed and undetermined.

### 2.3 The L-CG and N-CG method

The implementation of CG in image reconstruction field is generally in two ways. CG is one of the most useful methods for solving large linear systems of equations with symmetric and positive definite parameters, as it is called L-CG [19]. L-CG can be employed in FMT reconstruction by transforming equation (1) into a standard linear system. Since all parameters of each step in L-CG can be obtained from the value of the last step by iterative functions, the computation and storage of the algorithm are reduced. Besides, with pertinence, L-CG converges fast and has a good orientating ability. However, it is brittle and sensitive to noise. The requirement of the standard form of the problem in L-CG limits the implementation of the regularization and penalty methods, which are quite important for the illposed problem in FMT reconstruction. Thus, the CG method for nonlinear optimization problems, namely N-CG, which is more flexible to work along with the regularization and penalty methods and has a better capability to work under noise, is used widely for image reconstruction [15, 17]. According to the least-squares (LS) rule, problem (3) can be changed into a nonlinear optimization problem as follows:

(4) $$\min\phi (x)=\frac{1}{2}∥I-Wx{∥}^{2}+\eta (x),$$

where *η*(**x**) is the regularization or penalty term chosen on various purposes. Then the N-CG method can be adopted to find the optimal solution of (4). However, defects exist in N-CG. This method is more computationally expensive than L-CG, resulting in more time consuming for each iteration. Besides, it converges slowly [20]. Nevertheless, it is noticed that the properties of N-CG and L-CG are reciprocal. Thus, combining N-CG and L-CG together may generate an improved algorithm, which can get a higher speed and accuracy from L-CG as well as a good antinoise capability and the flexibility from N-CG. Therefore, an improved CG-based algorithm for FMT reconstruction, penalized linear and nonlinear combined conjugate gradient method (PLN-CG), was developed according to this consideration. The main scheme of the algorithm is presented in the following section.

## 3. A PENALIZED LINEAR AND NONLINEAR COMBINED CG METHOD

### 3.1. Searching the rough region using L-CG

**Figure 1:**
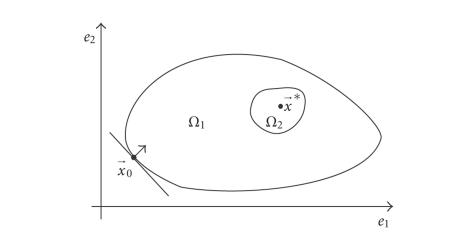
The sketch of the combined L-CG and N-CG schemes.

The searching process for the optimal solution x* in PLN-CG begins with an initial guess **x**_0_, and takes a steepest descent first step. The sketch of the scheme is shown in Figure 1.

At first, the search is general and the effect of noise is low, so L-CG is employed to find the rough region of the optimal solution x*, that is, Ω_2_. Because L-CG has a better orientating ability, and needs less computation, it can find Ω_2_ faster and more accurately, while it does not have to expose its fragility under noise.

Transformation has to be made to (3) to make it a standard linear system with symmetric positive definite coefficient matrix. The optimal solution of the LS problem described in (4) satisfies the normal equation as follows:

(5) **W**^*T*^**W***x* = **W**^*T*^**I**,

where **W** ^*T*^ is the transpose of **W**. Thus

(6) W*x = I* ,

where W* = **W** ^*T*^ **W**, is an *N* × *N* symmetric matrix. The reconstruction problem has become a standard linear one, as is required by L-CG.

Starting from an initial guess **x**_0_, the solution can be updated iteratively by

(7) **x**_*k+1*_ = **x**_*k*_ + *α*_*k*_**P**_*k*_,

where *α*_*k*_ is the step size

(8) $$\alpha_{k}=\frac{r_{k}^{T}r_{k}}{p_{k}^{T}W^{*}p_{k}},$$

and **r**_*k*_ is the gradient of each step. It is defined in L-CG as the residue of the linear system, which is obtained iteratively by

(9) **r**_*k+1*_ = **r**_*k*_ + *α*_*k*_W*P_*k*_,

where **p** *_k_* is the searching direction and

(10) $$\begin{array}{c} p_{k+1}=-r_{k+1}+\beta_{k+1}p_{k},\\ \beta_{k+1}=\frac{r_{k+1}^{T}r_{k+1}}{r_{k}^{T}r_{k}}. \end{array}$$

The L-CG searching iteration process will cease when **x**_*k*_ enters the region Ω_2_. The definition of the region Ω_2_ is determined by a restarting parameter, which is described in the following section.

### 3.2. The restart strategy

The restart strategy is a modification that is often used in nonlinear conjugate gradient procedures [19, 21]. The general scheme is to restart the iteration and take a steepest descent step according to some predetermined conditions. Restarting serves to periodically refresh the algorithm, erase old information that may not be beneficial or even harmful, and renew the initial guess **x**_0_ at every restarting time for the new iteration process.

We adopt a restart strategy in the PLN-CG scheme described as follows:

(11) |**r**_*k*_| = |**r**_*k* −1 _+ α_*k* −1_W*P_*k*−1_ | ≤ δ ,

where **r**_*k*_ represents the gradient of *ϕ*(**x**_*k*_). When |**r**_*k*_| satisfies (11), it means that the **x**_*k*_ obtained at current iteration has entered the small region Ω_2_ around x*. Then, a steepest descent step is taken, using the gradient direction at current point as the searching direction. At the same time, a new iteration process with the N-CG method begins, using **x** *_k_* as the initial guess **x**_0_. The experiential typical value for *δ* is between 10^−3^ and 10^−5^. Normally, we choose 10^−4^ for practical use.

#### 3.3. Use of the N-CG method

After entering Ω_2_, the searching result is getting quite closer to the optimal solution, so the effect of noise has to be taken into consideration. Besides, the uncertainty of the searching has increased. Thus, the method has been shifted to N-CG, which can work better with noisy data. Besides, N-CG can introduce the penalty or regularization method to gain a constraint as well as to reduce the illposedness.

Now, problem (3) is transformed into a nonlinear optimization problem:

(12) min ⁡ϕ(x)=12∥I−Wx∥2+η(x),

where *η*(**x**) is a penalty term which will be discussed in Section 3.4 .

The N-CG method differs from L-CG mainly in two ways. Firstly, rather than using a standard iterative function to find the step length *α*_*k*_, a line search method is used to identify an approximate minimum of the nonlinear function *ϕ*(**x**) along the searching direction **p** *_k_* [15, 17, 19]. Secondly, the gradient of *ϕ*(**x**) in L-CG is simply the residue of the linear system that can be obtained iteratively. While for N-CG, it must be replaced by the gradient of the nonlinear objective *ϕ*(**x**), that is, ∇*ϕ*(**x**).

Thus, using the **x**_*k*_ obtained from L-CG as the initial guess **x**_0_ for N-CG, the solution is updated iteratively:

(13) **x**_*k*+1_ = **x**_*k*_ + α_*k*_**P**_*k*_,

where *α*_*k*_ is the step size that is computed by a line search method,

(14) min *f* (**x**_*k*_ + α**P**_*k*_) s.t. α ≥ 0,

where **p** *_k_* is the searching direction and

(15) $$\begin{array}{l} p_{k+1}=-r_{k+1}+\beta_{k+1}p_{k},\\ \beta_{k+1}=\frac{r_{k+1}^{T}(r_{k+1}-r_{k})}{r_{k}^{T}r_{k}}, \end{array}$$

where **r**_*k*_ is the gradient of the objective function *ϕ*(**x**) at current point, that is,

(16) **r**_*k*_ = ∇ ϕ (**x**_*k*_).

#### 3.4. The nonnegative penalty

It is known that a major problem of the conventional gradient-based methods is that they are mainly designed for unconstrained problems, but the fluorescent source distribution in the biological tissue has to be constrained to a nonnegative region [16, 22]. Here, a quadratic penalty method [15, 19] is adopted to give the problem a nonnegative constraint.

Consider the penalty function described below

(17) $$\eta =\gamma \sum_{i}x_{i}^{2}u(-x_{i}),$$

where *x*_*i*_ is the *i*th element of **x**, *u*(*x*) is the unit step function. During the searching procedure, when the searched result **x** at current iteration has negative values, the penalty term will be increased. In this way, it will penalize **x** and force it to go back. *γ* is a penalty weighting parameter, which will gradually become zero as the iteration number increases. Thus, the solution of the new unconstrained problem in (12) with the penalty term (17) will approach the solution of the original problem in (3). The value of *γ* will be discussed experimentally in Section 4.1.3

Thus, a penalized linear and nonlinear combined conjugate gradient method is generated according to the scheme described above. The main flow of Algorithm 1 is listed below.

### 4. SIMULATIONS AND RESULTS

#### 4.1. Simulations with two sources

**Figure 2:**
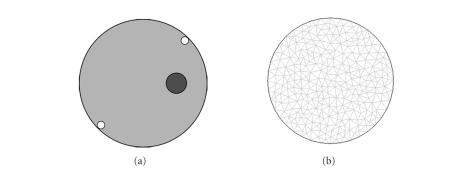
(a) Configuration of the simulation experiment using two excitation sources. The object is homogeneous, with a fluorophore (designated with •) imbedded in it. Two excitation sources (designated with ∘) are placed around the inner surface of the object. (b) Mesh in the forward FEM model.

In this experiment, a numerical model was set up to test the validity of the PLN-CG algorithm. A circular object was simulated with an outer diameter of 25 mm, which had a fluorophore with a diameter of 4 mm embedded in it. We supposed the optical property to be homogeneous, with *μ*_*a*_= 0.005mm ^−1^ and *μ*_*s*_= 1 mm^−1^ . In order to show the efficiency of PLN-CG better, only two excitation sources were used this time. They were placed around the inner surface of the circular object (as shown in Figure 2(a)), and were turned on in turn. For each source, 32 detector readings were available through the detector fibers, which were distributed uniformly on the surface of the circular object.

The forward data were simulated by finite element method [6, 10], using a FEM light transport model in CW mode [7]. The object was divided into 518 small triangular elements and the mesh is shown in Figure 2(b). The FEM forward engine was based on COMSOL Multiphysics (Section 3.2). The reconstruction algorithm was programed in MATLAB 6.5. A computer with CPU AMD Athlon ×23600+ and 512M DDRII memory was used.

**Figure 3:**
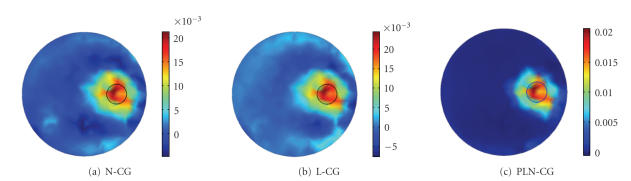
Images reconstructed with different methods. (a) N-CG, (b) L-CG, (c) PLN-CG. All results were obtained with a hundred iterations, *γ* was chosen to be 50. A zero vector was used as the initial guess. The small circle in each figure shows the real distribution of the fluorophore.

Images reconstructed by N-CG, L-CG, and PLN-CG are shown in Figures 3(a), 3(b), and 3(c). All images were obtained with one hundred iterations, as the objective function would descend very slowly thereafter. The nonnegative penalty parameter *γ* used for PLN-CG was 50. A zero vector was used as the initial guess for each algorithm.

It can be seen that images reconstructed by N-CG and L-CG are noisy. Negative values exist, which affect the accuracy of the results. While for PLN-CG, the values are all nonnegative, and the image is cleaner and more accurate. The computing time was about 5.02 seconds for N-CG, 0.22 second for L-CG, and 1.45 seconds for PLN-CG. It indicates that L-CG is much faster than N-CG. So helping N-CG with L-CG has tremendously reduced the computing time, as in the PLN-CG method.

##### 4.1.1. Reconstruction using different initial guesses

Being sensitive to the initial guess is a big disadvantage for most of the iterative approach based algorithms. It is regarded as a standard to test the stability of the algorithm.

**Figure 4:**
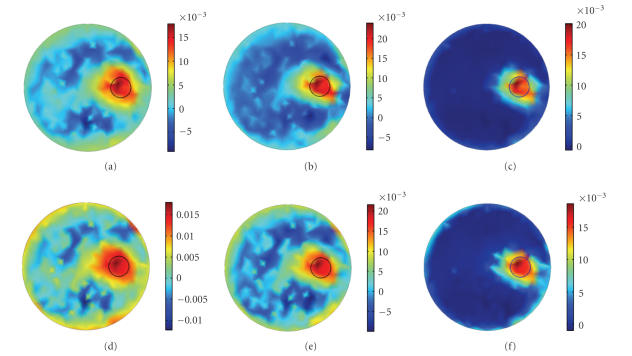
Images reconstructed with different initial guesses, using N-CG (Column 1), L-CG (Column 2), and PLN-CG (Column 3). Initial guess for (a)–(c) was an all-0.005 vector, and for (d)–(f) was an all-0.01 vector. Results were all obtained with one hundred iterations. *γ* was 50 for the PLN-CG approach. The small circle in each figure shows the real distribution of the fluorophore.

Figure 4 shows the results reconstructed with different initial values, using N-CG (Column 1), L-CG (Column 2), and PLN-CG (Column 3), respectively. Since most elements of the original solution are zero and the quantity of the fluorochrome intensity in FMT is relatively small, a zero vector is closer to the solution of the problem and is a better choice to be the initial value (Figure 3). When the initial value is increased to 0.005 and 0.01, the reconstructed images of N-CG (Figures 4(a) and 4(d)) and L-CG (Figures 4(b) and 4(e)) become perturbed, with artifacts distributed in the background. Whereas the PLN-CG (Figures 4(c) and 4(f)) is still giving a clear result, with only a slight blur on the edge.

##### 4.1.2. Reconstruction using noisy data

**Figure 5:**
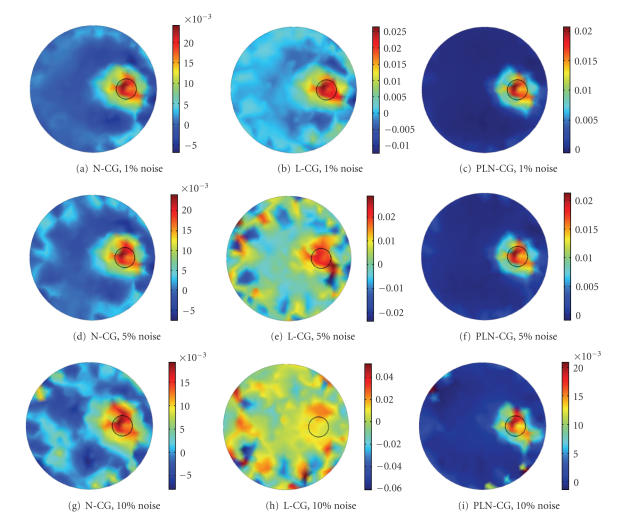
Images reconstructed with noisy data, using N-CG (Column 1), L-CG (Column 2), and PLN-CG (Column 3). Noise level for (a)-(c) was 1%, for (d)–(f) was 5%, and for (g)–(i) was 10%. Results reconstructed with N-CG and L-CG were obtained with one hundred and fifty iterations. For PLN-CG, when *γ*was 50, the iteration number was one hundred. The small circle in each figure shows the real distribution of the fluorophore.

To test the stability of the algorithm, white Gaussian noise was added to the detector readings. Figure 5 shows the images reconstructed by N-CG (Column 1), L-CG (Column 2), and PLN-CG (Column 3). The L-CG method reveals its fragility under noise. The image is perturbed when the noise level is 5% (Figure 5(e)). When the noise level is 10%, the image is totally blurred, as is shown in Figure 5(h). The N-CG method has a better performance compared with L-CG (Figures Figure 5(i) and Figure 5(g)). However, many artifacts exist in the images and affect the quantification of the fluorophore. Whereas, images reconstructed with PLN-CG approach are clear when the noise levels are 1% (Figure 5(c)) and 5% (Figure 5(f)). When the noise level is 10%, the fluorescent source distribution is still relatively clear, with a little artifacts appearing on the edge.

##### 4.1.3. The value of the penalty parameter *γ*

**Figure 6:**
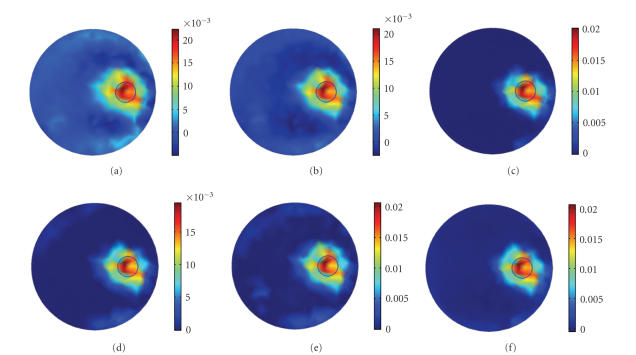
Reconstructed images using different *γ* : (a) *γ* = 1*e* −3; (b) *γ* = 1; (c) *γ* = 1*e*3; (d) *γ* = 1*e*5; (e) *γ* = 1*e*7; (f) *γ* = 0.005*n* ^2^, where *n* is the iteration number. A zero vector was used as the initial guess for each reconstruction process, and all results were obtained with a hundred iterations. The small circle in each figure shows the real distribution of the fluorophore.

When using the PLN-CG method, *γ* is the weighting parameter that controls the effect of the penalty term. Figure 6 shows the images reconstructed with different *γ*.

It can be seen that when *γ* is 10^−3^, the effect of the penalty term is not enough. Negative values exist and the background is not clean. Increasing *γ* to 1 does produce better results (Figure 6(b)), and a further increase to 10^3^ enhances the improvement (Figure 6(c)). When *γ* increases to 10^5^, the quality of the image begins to get worse (Figure 6(d)). The results show that the penalty term can work well for a large variation of *γ*. A typical value for *γ* is 10 to 10^3^. Besides, *γ* should be increased when the total iteration number increases.

In addition, rather than keeping *γ* fixed, one can use different *γ* according to the experiential equation [18]

(18) *γ* = *an*^2^,

where *n* is the iteration number. *a* is a fixed weighting parameter, which can be set to a value between 10^−3^ and 1. Figure 6(f) shows the images reconstructed according to (18). The iteration number was one hundred and *a* was chosen to be 0.005.

### 4.2. Simulations with more sources

**Figure 7:**
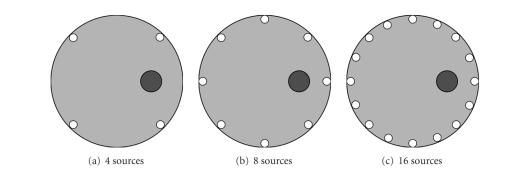
Configuration of the simulation experiments using more excitation sources. (a) 4 sources. (b) 8 sources. (c) 16 sources. The excitation sources are distributed uniformly around the inner surface of the object (designated with ∘). For each experiment, the object is homogeneous, with a fluorophore (designated with •) embedded in it.

Simulation studies above were based on two excitation sources, in order to demonstrate the qualities of the PLN-CG approach better. When the number of sources is increased, a larger dataset can be obtained. It will improve the information content of the measurements and reduce the illposedness of the inverse problem [5]. Thus, in practice, FMT equipments normally use more excitation sources [3, 4]. Here, simulation experiments were designed using 4 sources (Figure 7(a)), 8 sources (Figure 7(b)), and 16 sources (Figure 7(c)), respectively.

**Figure 8:**
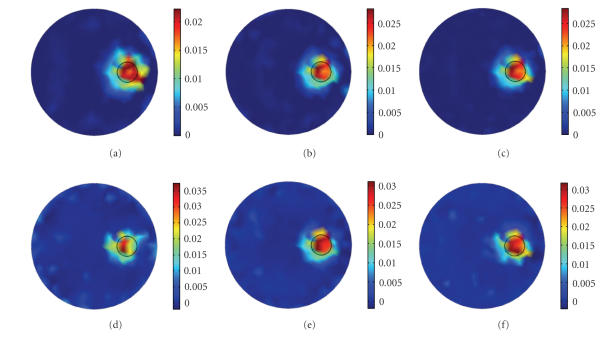
Images reconstructed in the experiments which have more excitation sources. (a) 4 sources, clean data; (b) 8 sources, clean data; (c) 16 sources, clean data; (d) 4 sources, 10% noise; (e) 8 sources, 10% noise; (f) 16 sources, 10% noise. The small circle in each figure shows the real distribution of the fluorophore.

In each experiment, sources were turned on in turn and 32 detector readings were available for each source. Results with clean data were obtained with a hundred iterations for about 2.99 seconds in the 4 sources case (Figure 8(a)). While the computing time was about 4.9220 seconds and 9.1560 seconds for 150 iterations in the 8 sources case (Figure 8(b)) and 16 sources case (Figure 8(c)), as they have a larger dataset. *γ* was simply set to 50 for all cases because the difference among the iteration numbers was small. It is shown that as the source number increases, the qualities of the reconstructed images are in progress. The reconstructed fluorochrome region marked with the small black circle is more even and closer to the original value.

After the experiments using clean data described above, white Gaussian noise with a constant variance was added to the detector readings. The noise level was 10%. It is shown that the reconstructed results become clearer and better when the sources number increases from 2 (Figure 5(i)) to 4 (Figure 8(d)) and 8 (Figure 8(e)). However, when using 16 sources (Figure 8(f)), the image is not improved compared with the 8 sources case, or even worse, which defies the common sense. The reason may be that, when using clean and accurate data for the reconstruction, more datasets mean more information, whereas for the cases using noisy data, too many data may interfere with each other and counteract the effect. Nevertheless, the results of this experiment further demonstrate the capability of the PLN-CG method to work under noise.

## 5. DISCUSSION AND CONCLUSION

The goal of this work was to establish a fast and accurate algorithm for FMT reconstruction, which is illposed. In order to achieve this goal, a penalized linear and nonlinear combined conjugate gradient algorithm was developed. Simulation studies have indicated that this PLN-CG method can exhibit very favorable performance and produce relatively stable behavior. Further studies show that, when using sixteen sources, the reconstruction algorithm can work under 15% noise, which is sufficient for practical use. The better performance is partly achieved by the combination of L-CG and N-CG. L-CG makes the algorithm faster and more accurate. While at the same time, N-CG gives the whole algorithm a better capacity to deal with noise. It introduces the penalty method to get a nonnegative constraint and reduce the uncertainty of the problem. The restart strategy also improves the efficiency of the algorithm by refreshing the algorithm periodically.

Further improvement can be made for the PLN-CG algorithm in future. Some kind of regularization techniques can be employed to regularize the results and smoothen the images [6]. The prior knowledge about the intensity of the fluorochrome can be used to utilize a general threshold of the reconstructed fluorescent source density to decrease the permissible region [11]. In addition, doing more restarting procedures appropriately may also upgrade the reconstruction images. Currently, we are involved in the practical use of the PLN-CG reconstruction algorithm for the ongoing FMT experiment in our laboratory.

## Figures and Tables

**Algorithm 1:**
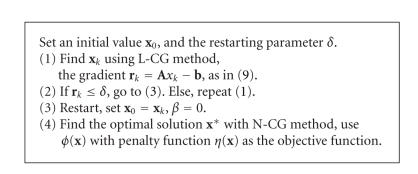
The PLN-CG scheme for FMT reconstruction.
